# Extracellular Signal-Regulated Kinase 1 Alone Is Dispensable for Hyperoxia-Mediated Alveolar and Pulmonary Vascular Simplification in Neonatal Mice

**DOI:** 10.3390/antiox11061130

**Published:** 2022-06-08

**Authors:** Renuka T. Menon, Shyam Thapa, Amrit Kumar Shrestha, Roberto Barrios, Binoy Shivanna

**Affiliations:** 1 Section of Neonatology, Department of Pediatrics, Baylor College of Medicine (BCM), Houston, TX 77030, USA; renuka.menon@bcm.edu (R.T.M.); shyam.thapa@bcm.edu (S.T.); amrit.shrestha@bcm.edu (A.K.S.); 2 Department of Pathology and Genomic Medicine, Houston Methodist Hospital, Houston, TX 77030, USA; rbarrios@houstonmethodist.org

**Keywords:** extracellular signal-regulated kinases 1 and 2, neonatal HPMECs, hydrogen peroxide, hyperoxia, bronchopulmonary dysplasia

## Abstract

Bronchopulmonary dysplasia (BPD) is a morbid lung disease distinguished by lung alveolar and vascular simplification. Hyperoxia, an important BPD causative factor, increases extracellular signal-regulated kinases (ERK)-1/2 expression, whereas decreased lung endothelial cell *ERK2* expression reduces angiogenesis and potentiates hyperoxia-mediated BPD in mice. However, ERK1′s role in experimental BPD is unclear. Thus, we hypothesized that hyperoxia-induced experimental BPD would be more severe in global *ERK1*-knockout (*ERK1*^-/-^) mice than their wild-type (*ERK1*^+/+^ mice) littermates. We determined the extent of lung development, ERK1/2 expression, inflammation, and oxidative stress in *ERK1*^-/-^ and *ERK1*^+/+^ mice exposed to normoxia (FiO_2_ 21%) or hyperoxia (FiO_2_ 70%). We also quantified the extent of angiogenesis and hydrogen peroxide (H_2_O_2_) production in hyperoxia-exposed neonatal human pulmonary microvascular endothelial cells (HPMECs) with normal and decreased *ERK1* signaling. Compared with *ERK1*^+/+^ mice, *ERK1*^-/-^ mice displayed increased pulmonary ERK2 activation upon hyperoxia exposure. However, the extent of hyperoxia-induced inflammation, oxidative stress, and interrupted lung development was similar in *ERK1*^-/-^ and *ERK1*^+/+^ mice. *ERK1* knockdown in HPMECs increased ERK2 activation at baseline, but did not affect in vitro angiogenesis and hyperoxia-induced H_2_O_2_ production. Thus, we conclude *ERK1* is dispensable for hyperoxia-induced experimental BPD due to compensatory ERK2 activation.

## 1. Introduction

Bronchopulmonary dysplasia (BPD) remains the most common lung complication of extremely preterm neonates despite significant advances in the medical care of these infants [1]. BPD is also the most expensive neonatal disease; the costs required to provide medical care for a BPD infant are double that of a non-BPD infant in the first year of life [2]. Further, BPD has long-lasting effects on the health of preterm infants and affects the psychosocial and emotional well-being of their parents [3,4,5,6,7].

The lungs of BPD infants have fewer alveoli and blood vessels BPD [8,9]. Several studies have consistently shown the importance and necessary role of healthy lung blood vessels for normal lung development and for the ability of the lungs to recover from insults efficiently [10,11,12,13]. The well-known angiogenic molecules, vascular endothelial growth factor (VEGF) and nitric oxide (NO), are required for lung development in health and disease in neonatal animals. VEGF mitigates experimental BPD and pulmonary hypertension (PH) via the endothelial nitric oxide synthase pathway [14,15,16]. However, neither prophylactic nor rescue inhaled NO therapies decreased the BPD burden in humans [17,18]. However, evidence from human studies suggests that using combination therapies can reduce the BPD burden [19]. Thus, we sought to investigate molecular targets complementing NO therapy to protect and advance lung vascular health.

Mitogen-activated protein (MAP) kinases are targets of growth factors that facilitate lung development [20]. Among these kinases, the extracellular signal-regulated kinase (ERK) 1/2 promotes proliferation and differentiation of many cell types, including the endothelial and epithelial cells, whereas c-Jun NH_2_-terminal kinases and p38 kinase induce cell apoptosis [21]. During development, ERK1/2 are active [22,23] and modulate morphogenesis in several organs, including the lungs [24,25,26]. Further, we showed that ERK1/2 is necessary for human lung microvascular endothelial cell (HPMEC) tubule formation, and hyperoxia activates these enzymes in neonatal HPMECs and murine lungs [27]. Using *Tie-2 Cre*-mediated decrease in lung endothelial cell *ERK2* expression, we also demonstrated that endothelial *ERK2* deficiency augments experimental BPD-associated PH [28]. However, the role of the *ERK1* gene in neonatal lung injury is not well studied. Therefore, we investigated if *ERK1* deficiency decreases angiogenesis and potentiates hyperoxic injury in neonatal murine lungs in this study. Because hyperoxia leads to BPD and the phenotypes of neonatal murine hyperoxia-induced lung injury and human BPD [29,30,31,32] are similar, we used our mouse hyperoxia model [32] to investigate the role of *ERK1* signaling in experimental BPD. We hypothesized that hyperoxia-induced experimental BPD would be more severe in global *ERK1*-knockout (*ERK1*^-/-^) mice than their wild-type (*ERK1*^+/+^ mice) littermates. We also used HPMECs to examine the contributory role of *ERK1* in angiogenesis and oxidative stress in developing human lungs.

## 2. Materials and Methods

### 2.1. In Vitro Experiments

#### 2.1.1. Cell Culture

Immortalized microvascular endothelial cells isolated from human neonatal lungs (HULEC-5a) were obtained from American Type Culture Collection (ATCC, Manassas, VA; CRL-3244) and grown in 95% air and 5% CO_2_ at 37 °C, as described recently [33]. We used cells between Passages 6 and 12 for our studies.

#### 2.1.2. Transfection Experiments

The HULEC-5a cells were transfected for up to 48 h with either 50 nM control siRNA (Dharmacon, Lafayette, CO, USA; d-001810) or 50 nM *ERK1* siRNA (Dharmacon; L-003592) using Lipofectamine RNAiMAX (Life Technologies, Grand Island, NY, USA; 13778075). We determined the effects of *ERK1* knockdown on ERK1/2 expression and activation, tubule formation, and hydrogen peroxide (H_2_O_2_) generation at the indicated time points.

#### 2.1.3. Exposure of Cells to Hyperoxia

Hyperoxia experiments were conducted in a plexiglass, sealed chamber into which a mixture of 70% O_2_ and 5% CO_2_ was circulated continuously using a ProOx 110 Compact O_2_ Controller (BioSpherix, Parish, NY, USA).

#### 2.1.4. Western Blot Assays

Cells were grown on six-well plates to 60% confluence and exposed to normoxia (21% O_2_ and 5% CO_2_) or hyperoxia (70% O_2_ and 5% CO_2_) for up to 48 h. In a separate set of experiments, cells grown on six-well plates to 60–70% confluence under normoxic conditions (21% O_2_ and 5% CO_2_) were transfected with control or *ERK1* siRNA for 48 h, as described above, after which whole-cell protein extracts were obtained and subjected to immunoblotting with the following primary antibodies: anti-phospho ERK1/2 (Cell Signaling, Danvers, MA, USA; 9106, dilution 1:1000), anti-total ERK1/2 (Cell Signaling, Danvers, MA, USA; 4695, dilution 1:1000), and anti-vinculin (Cell Signaling Technology; 13901, 1:8000) antibodies. The immunoreactive bands were detected and quantified, as described before [34]. Vinculin was used as the reference protein.

#### 2.1.5. Tubule Formation Assay

Tubule formation was determined by Matrigel assay [35,36]. Briefly, HULEC-5a cells transfected with control or *ERK1* siRNA and exposed to normoxia (21% O_2_ and 5% CO_2_) or hyperoxia (70% O_2_ and 5% CO_2_) for 48 h were grown in 15-well µ-slide microplates (Ibidi, Gräfelfing, Germany; 81506) containing growth-factor-reduced Matrigel (Corning, New York, NY, USA; 356230) at a density of 8 × 10^3^ cells per well. We quantified the tubule number following an 18 h incubation period by imaging with a 4× objective using the Keyence microscope (Keyence Corporation, Itasca, IL, USA). The tubule number was quantified using the Image J software (version 1.8; https://imagej.nih.gov, accessed on 1 June 2022; National Institutes of Health, Bethesda, MD, USA).

#### 2.1.6. Measurement of H_2_O_2_ Generation

The H_2_O_2_ production was quantified by the ROS-Glo™ H_2_O_2_ Assay (Promega, Madison, WI, USA; G8820) according to the manufacturer’s protocol. Briefly, cells grown on 96-well plates to 60–70% confluence were transfected with 50 nM control or *ERK1* siRNA for 24 h. The siRNA-transfected cells were then exposed to normoxia (21% O_2_ and 5% CO_2_) or hyperoxia (70% O_2_ and 5% CO_2_) for 24 h. Six hours prior to the completion of hyperoxia exposure experiments, the H_2_O_2_ substrate was added to the wells, and the cell culture plates were returned to the incubator for the remainder of the experiment. At the end of the experiments, the cells were incubated with ROS-Glo™ detection solution for 20 min at room temperature before the relative luminescence units were measured using the Spectramax M3 luminescence microplate reader.

#### 2.1.7. Statistical Analyses

We analyzed the results using the GraphPad Prism 9 software. The experiments were repeated at least more than one time. Data are expressed as mean ± SD. The differential effects of *ERK1* siRNA transfection on the outcomes of interest were determined by *t*-test or ANOVA. A value of *p* < 0.05 was considered significant.

### 2.2. In Vivo Experiments

#### 2.2.1. Animals

This study was approved and conducted in strict accordance with the federal guidelines for the humane care and use of laboratory animals by the Institutional Animal Care and Use Committee of Baylor College of Medicine (Protocol Number: AN-5631). *ERK1^-/-^* mice (B6.129 (Cg)-Mapk3tm1Gela/J, 019113) were obtained from the Jackson Laboratory (Bar Harbor, ME, USA). Timed-pregnant mice raised in our animal facility were used for the experiments. We determined the genotype of the mice, as recommended by the Jackson Laboratory.

#### 2.2.2. Hyperoxia Experiments

*ERK1*-sufficient (*ERK1^+/+^*) or -knockout (*ERK1*^-/-^) mice were exposed to normoxia (FiO_2_ 21%) or hyperoxia (FiO_2_ 70%) from postnatal day (PND) 1 to PND14, as described before [37]. We rotated the dams between normoxia- and hyperoxia-exposed litters every 24 h during our study to avoid oxygen toxicity in the dams.

#### 2.2.3. Analyses of Lung Alveolarization and Vascularization

On PND14, we inflated and fixed the lungs with 4% paraformaldehyde, as mentioned previously. Paraffin-embedded lung sections were obtained for the analysis of lung alveolarization and vascularization [37]. Alveolarization was determined by radial alveolar counts (RAC) and mean linear intercepts (MLI), whereas pulmonary vessel density was determined by quantifying von Willebrand factor (vWF) stained blood vessels less than 150 µm in diameter [32].

#### 2.2.4. Lung Tissue Extraction for Genomic and Proteomic Studies

The lungs were snap-frozen in liquid nitrogen on PND7 and stored at −80 °C for subsequent RNA and protein studies. Total lung RNA was extracted and reverse transcribed to cDNA [38]. Whole lung protein was extracted as follows: the lung tissue was homogenized with a mortar and pestle in T-PER™ Tissue Protein Extraction Reagent (Thermo Fisher Scientific; 78,510) with Halt™ Protease and Phosphatase Inhibitor Cocktail (Thermo Fisher Scientific; 78,442). The homogenate was centrifuged at 10,000× *g* for 5 min at 4 °C, and the supernatant, protein lysate, was stored at −80 °C.

#### 2.2.5. Real-Time RT- PCR Assays

Real-time quantitative RT-PCR analysis was done using the following TaqMan gene specific primers: *chemokine (C-C motif) ligand 2* (*CCL2*; Mm00441242_m1), *CCL3* (Mm00441259_g1); *ERK1* (Mm01973540_g1); *ERK2* (Mm00442479_m1); *heme oxygenase 1* (*HO1*; Mm00516005_m1), intercellular adhesion molecule-1 (*ICAM-1;* Mm00516023_m1), *interleukin (IL)-1β* (*IL-1β*; Mm00434228_m1), *IL-10* (Mm01288386_m1); *NAD(P)H quinone dehydrogenase 1* (NQO1; Mm01253561_m1), *tumor necrosis factor-α* (*TNF-α*; Mm00443258_m1), *glutathione peroxidase 2* (*GPX2;* Mm00850074_g1); and *glyceraldehyde 3-phosphate dehydrogenase* (*GAPDH*; Mm99999915_g1).

#### 2.2.6. Western Blot Assays

Immunoblotting assays were performed as described above using the following primary antibodies: anti-phospho ERK1/2 (Cell Signaling, Danvers, MA, USA; 9106, dilution 1:1000), anti-total ERK1/2 (Cell Signaling, Danvers, MA, USA; 4695, dilution 1:1000), anti-SOD1 (Santa Cruz Biotechnologies; Santa Cruz, CA, sc-8637, dilution 1:1000), anti-SOD2 (Santa Cruz Biotechnologies; sc-137254, dilution 1:1000), and anti-vinculin (Cell Signaling Technology; 13901, 1:8000) antibodies.

#### 2.2.7. Statistical Analyses

GraphPad Prism 9 software was used to analyze the results. The experiments were repeated at least more than one time. Data are expressed as mean ± SD. The individual and interactive effects of *ERK1* gene expression and hyperoxia exposure on lung inflammation, antioxidant enzyme expression, alveolarization, and pulmonary vascularization were assessed using analysis of variance (ANOVA). A *p* value of < 0.05 was considered significant.

## 3. Results

**Figure 1:**
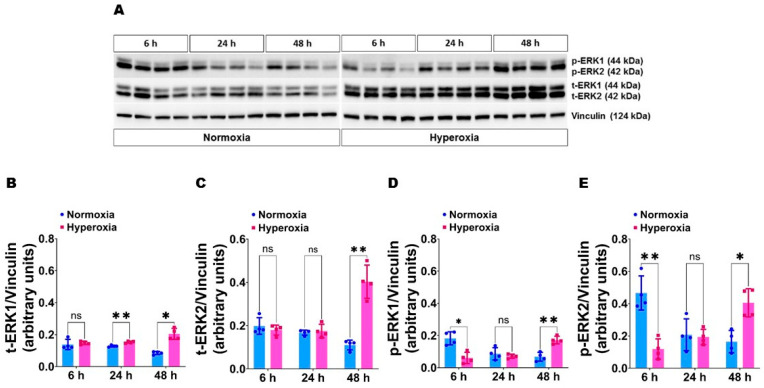
ERK1 and ERK2 expression and activation in HULEC-5a at baseline and following hyperoxia exposure. Cells grown on six-well plates to 60–70% confluence were exposed to normoxia (21% O_2_ and 5% CO_2_) or hyperoxia (70% O_2_ and 5% CO_2_) for up to 48 h. The cells were then harvested for protein expression studies at 6 h, 24 h, and 48 h of exposure. (**A**) Determination of total and phosphorylated ERK1 and ERK2 protein levels by immunoblotting. (**B**–**E**) Quantification and normalization of total (t)-ERK1 (**B**) and t-ERK2 (**C**) band intensities and phospho (p)-ERK1 (**D**) and p-ERK2 (**E**) band intensities to those of vinculin. Values represent the mean ± SD (*n* = 4/group). Significant differences between exposures at different time points are indicated by *, *p* < 0.05 and **, *p* < 0.01 (ANOVA). ns = not significant.

Expression and activation of ERK1 and ERK2 in HULEC-5a at baseline and following hyperoxia exposure: We initially sought to determine the relative expression and activation of ERK1 and ERK2 protein at baseline and following hyperoxia exposure in transformed human lung microvascular endothelial cells derived from a neonate (HULEC-5a) because hyperoxia and pulmonary microvascular endothelial cells play crucial roles in BPD pathogenesis. Our immunoblotting analyses suggest that the expression and activation of ERK2 protein (Figure 1A,C,E) are 1.3-fold to 2.5-fold greater than that of ERK1 (Figure 1A,B,D) at baseline in human pulmonary microvascular endothelial cells. Following hyperoxia exposure, the activation of ERK1 and ERK2 decrease initially at 6 h, followed by an increase at 48 h (Figure 1A–E). Consistent with the findings at baseline, the expression and activation of ERK2 (Figure 1A,C,E) were 1.2-fold to 2.7-fold greater than that of ERK1 (Figure 1A,B,D) following hyperoxia exposure.

**Figure 2:**
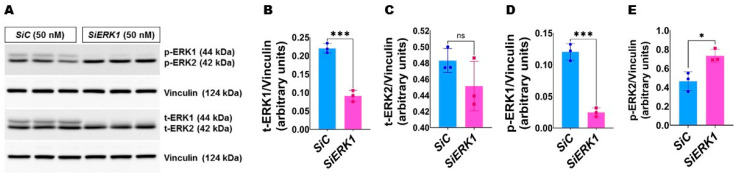
Effects of *ERK1* knockdown on ERK1/2 expression and activation in HULEC-5a. Cells grown on six-well plates to 60% confluence were transfected with 50 nM control or *ERK1* siRNA and exposed to normoxia (21% O_2_ and 5% CO_2_) for 48 h. The transfected cells were then harvested for protein expression studies. (**A**) Determination of total and phosphorylated ERK1 and ERK2 protein levels by immunoblotting. (**B**–**E**) Quantification and normalization of total (t)-ERK1 (**B**) and t-ERK2 (**C**) band intensities and phospho (p)-ERK1 (**D**) and p-ERK2 (**E**) band intensities to those of vinculin. Values represent the mean ± SD (*n* = 3/group). Significant differences between transfected cells are indicated by *, *p* < 0.05 and ***, *p* < 0.001 (*t*-test). ns = not significant.

ERK1 knockdown increases ERK2 activation in HULEC-5a: We next used HULEC-5a to investigate the effects of *ERK1* deficiency in hyperoxic lung injury. We used *ERK1* siRNA to knockdown ERK1 protein. *ERK1* siRNA reduced ERK1 protein expression and activation by greater than 2-fold to 7-fold (Figure 2A,B,D). *ERK1* knockdown increased ERK2 activation at basal conditions, as evidenced by increased phosphorylated ERK2 protein levels in *ERK1*-deficient cells (Figure 2A,E).

**Figure 3:**
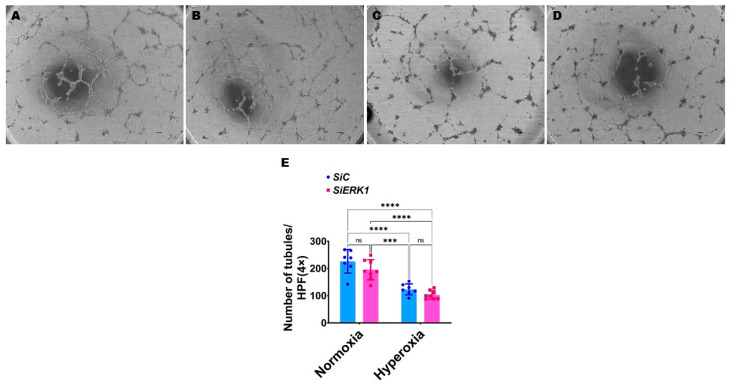
Effects of *ERK1* knockdown on HULEC-5a tubule formation. Cells grown on six-well plates to 60% confluence were transfected with control or *ERK1* siRNA and exposed to normoxia (21% O_2_ and 5% CO_2_) or hyperoxia (70% O_2_ and 5% CO_2_) for 48 h. The transfected cells were then harvested for tubule formation assay. (**A**–**D**) Representative photographs showing tubule formation of cells transfected with control (**A**,**C**) and *ERK1* (**B**,**D**) siRNA and exposed to normoxia (**A**,**B**) or hyperoxia (**C**,**D**). Scale bar = 100 µm. (**E**) Quantification of tubule formation. Values are presented as mean ± SD (*n* = 7–8/group). Significant differences between exposures are indicated by ***, *p* < 0.001 and ****, *p* < 0.0001 (ANOVA). ns = not significant.

ERK1 is not required for HULEC-5a tubule formation: Our previous studies demonstrated that pharmacological inhibition of *ERK1/2* signaling [27] and genetic inhibition of *ERK2* signaling [28] decrease human pulmonary microvascular endothelial cell tubule formation. So, we investigated if *ERK1* signaling is necessary for human neonatal lung angiogenesis by performing the tubule formation assay using normoxia (21% O_2_ and 5% CO_2_) and hyperoxia (70% O_2_ and 5% CO_2_) exposed *ERK1*-sufficient and -deficient HULEC-5a cells. Unlike genetic inhibition of *ERK2* signaling, knockdown of *ERK1* did not decrease HULEC-5a tubule formation either in normoxic or hyperoxic conditions (Figure 3).

**Figure 4:**
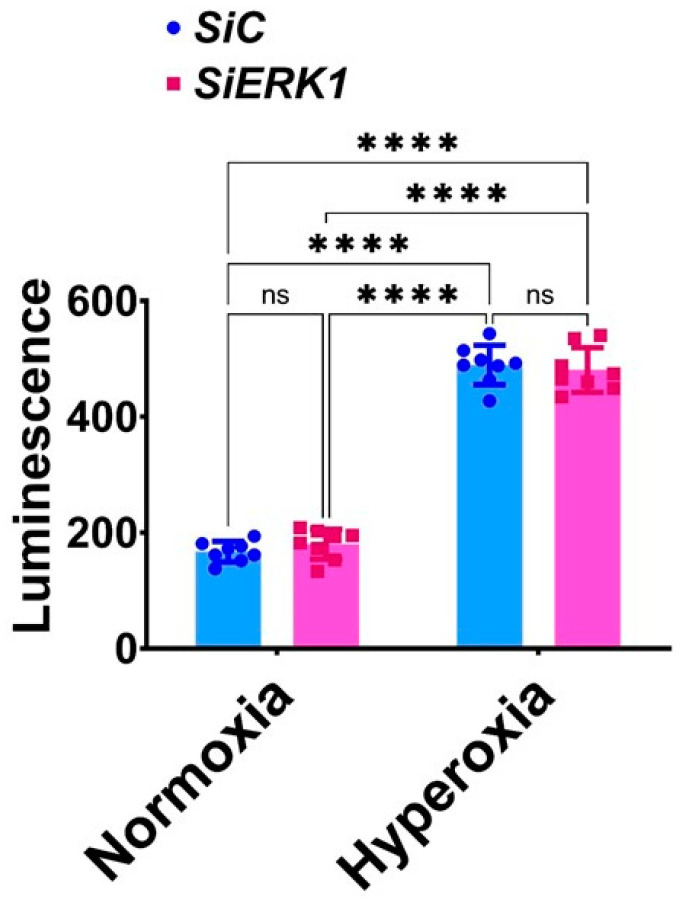
Effects of ERK1 knockdown on H_2_O_2_ generation in HULEC-5a. Cells grown on 96-well plates to 60–70% confluence were transfected with control or *ERK1* siRNA for 24 h, before being exposed to normoxia (21% O_2_ and 5% CO_2_) or hyperoxia (70% O_2_ and 5% CO_2_) for another 24 h. The transfected and exposed cells were then harvested for ROS-Glo™ H_2_O_2_ assay and quantified. Values represent the mean ± SD (*n* = 8/group). Significant differences between exposures are indicated by ****, *p* < 0.0001 (ANOVA). ns = not significant.

Knockdown of ERK1 does not potentiate hyperoxia-induced H_2_O_2_ generation in HULEC-5a: Reactive oxygen species (ROS) such as H_2_O_2_ are widely implicated in BPD pathogenesis. So, we investigated the effects of decreased *ERK1* signaling on H_2_O_2_ generation in our in vitro model of hyperoxic lung injury. In normoxic conditions, H_2_O_2_ levels were comparable between *ERK1*-sufficient and -deficient cells (Figure 4), indicating that the knockdown of *ERK1* does not affect H_2_O_2_ generation at baseline. Hyperoxic exposure increased H_2_O_2_ generation; however, the extent of this hyperoxia effect was similar in *ERK1*-sufficient and -deficient cells (Figure 4), suggesting that *ERK1* is not a major regulator of H_2_O_2_ generation even in hyperoxic conditions in HULEC-5a cells.

**Figure 5:**
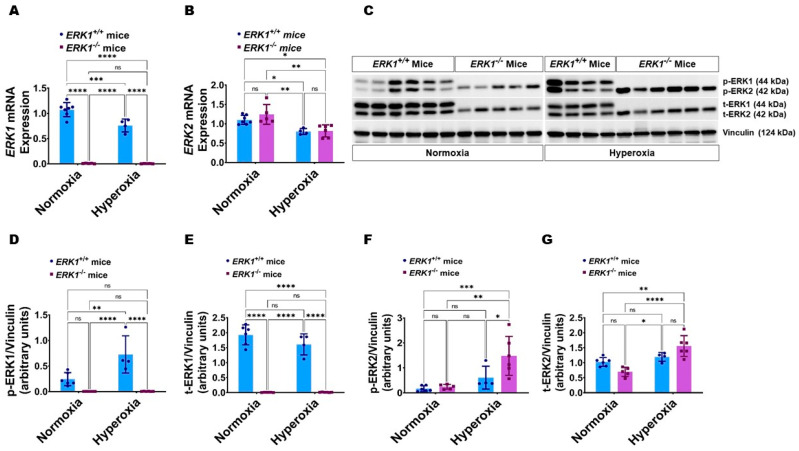
ERK1 and ERK2 expression and activation in the lungs of global *ERK1*-sufficient (*ERK1^+/+^*) and -knockout (*ERK1*^-/-^) mice. The gene and protein expression studies were done on PND7 following exposure of *ERK1^+/+^* or *ERK1*^-/-^ mice to either 21% O_2_ (normoxia) or 70% O_2_ (hyperoxia) from PND to PND7. (**A**,**B**) RT-PCR analyses-based determination of *ERK1* (**A**) and *ERK2* (**B**) mRNA levels. (**C**). Determination of total and phosphorylated ERK1 and ERK2 protein levels by immunoblotting. (**D**–**G**): Quantification and normalization of phospho (p)-ERK1 (**D**), total (t)-ERK1 (**E**), p-ERK2 (**F**), and t-ERK2 (**G**) band intensities to those of vinculin. Values are presented as mean ± SD (*n* = 4–7/genotype/exposure). Significant differences between genotypes and exposures are indicated by *, *p* < 0.05, **, *p* < 0.01, ***, *p* < 0.001, and ****, *p* < 0.0001 (ANOVA). ns = not significant.

Lung ERK2 activation is increased in global ERK1-knockout (ERK1^-/-^) mice: We recently observed that *ERK2* deficiency increases ERK1 activation in fetal HPMECs [28]. Therefore, we initially determined whether *ERK1*^-/-^ mice display increased pulmonary ERK2 activation than *ERK1^+/+^* mice. ERK1 mRNA (Figure 5A) and protein levels (Figure 5C–E) were unmeasurable in the lungs of *ERK1*^-/-^ mice. Further, while the pulmonary ERK2 mRNA (Figure 5B) and ERK2 protein expression and activation (Figure 5C,F,G) were comparable between *ERK1^-/-^* and *ERK1^+/+^* mice at basal conditions, the pulmonary phosphorylated ERK2 protein levels (Figure 5C,F) were greater in *ERK1*^-/-^ mice than in *ERK1^+/+^* mice upon hyperoxia exposure. These findings indicate that global *ERK1*^-/-^ mice display compensatory overactivation of ERK2 in our experimental conditions.

**Figure 6:**
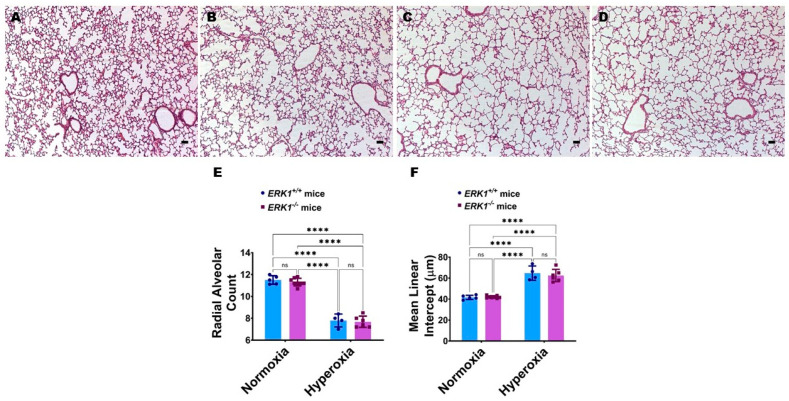
Lung alveolar development in global *ERK1*-sufficient (*ERK1^+/+^*) and -knockout (*ERK1*^-/-^) mice. Alveolarization was quantified on PND14 in *ERK1^+/+^* or *ERK1*^-/-^ mice exposed to either 21% O_2_ (normoxia) or 70% O_2_ (hyperoxia) from PND1 to PND14. (**A**–**D**) Representative hematoxylin and eosin-stained lung sections from *ERK1^+/+^* (**A**,**C**) and *ERK1*^-/-^ (**B**,**D**) mice exposed to normoxia (**A**,**B**) or hyperoxia (**C**,**D**). Scale bar = 100 µm. (**E**,**F**) Alveolarization was quantified by determining RAC (**E**) and MLI (**F**). Values are presented as the mean ± SD (*n* = 4–8/genotype/exposure). Significant differences between genotypes and exposures are indicated by ****, *p* < 0.0001 (ANOVA). ns = not significant.

Global ERK1 deficiency does not potentiate hyperoxia-induced alveolar simplification in neonatal mice: We investigated if the absence of the *ERK1* impacts lung alveolarization by analyzing radial alveolar counts (RAC) and mean linear intercepts (MLI). Hyperoxia reduced RAC (Figure 6A,C,E) while increasing the MLI (Figure 6A,C,F), suggesting that there were fewer and larger alveoli, respectively, in hyperoxia-exposed mice. However, the extent of hyperoxia-induced alveolar simplification was comparable between *ERK1*^-/-^ and *ERK1^+/+^* mice (Figure 6C–F), indicating that isolated *ERK1* deficiency does not potentiate or attenuate hyperoxia-induced alveolar simplification.

**Figure 7:**
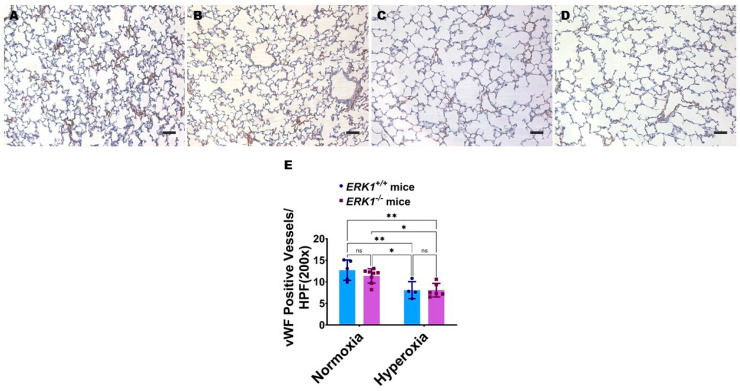
Lung vascular development in global *ERK1*-sufficient (*ERK1^+/+^*) and -knockout (*ERK1*^-/-^) mice. Lung vascularization was quantified on PND14 in *ERK1^+/+^* or *ERK1*^-/-^ mice exposed to either 21% O_2_ (normoxia) or 70% O_2_ (hyperoxia) from PND1 to PND14. (**A**–**D**) Representative vWF-stained lung sections from *ERK1^+/+^* (**A**,**C**) and *ERK1*^-/-^ (**B**,**D**) mice exposed to normoxia (**A**,**B**) or hyperoxia (**C**,**D**). Scale bar = 100 µm. (**E**) Quantitative analysis of vWF-stained lung blood vessels per high-power field (HPF). Values are presented as the mean ± SD (*n* = 4–8/genotype/exposure). Significant differences between genotypes and exposures are indicated by *, *p* < 0.05 and **, *p* < 0.01 (ANOVA). ns = not significant.

Global ERK1 deficiency does not potentiate hyperoxia-induced pulmonary vascular simplification in neonatal mice: In addition to alveolar simplification, fewer and dysmorphic lung blood vessels, i.e., pulmonary vascular simplification, is a hallmark of experimental BPD. Neonatal mice exposed to hyperoxia displayed fewer von Willebrand factor (vWF)-stained lung blood vessels (Figure 7A,C,E). However, the extent of hyperoxia-induced pulmonary vascular simplification was comparable between *ERK1*^-/-^ and *ERK1^+/+^* mice (Figure 7C–E), indicating that isolated *ERK1* deficiency does not potentiate or attenuate hyperoxia-induced pulmonary vascular simplification.

**Figure 8:**
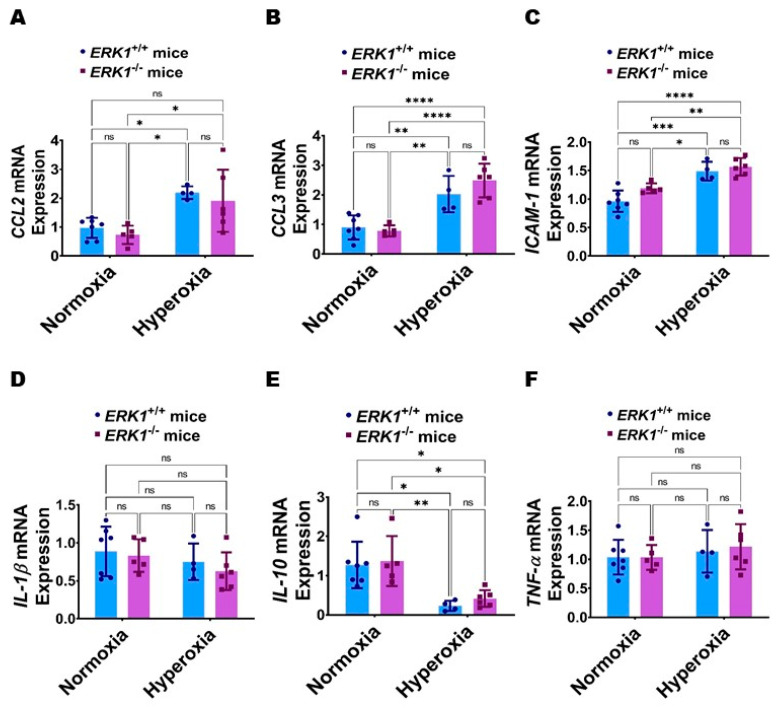
Lung inflammation in global *ERK1*-sufficient (*ERK1^+/+^*) and -knockout (*ERK1*^-/-^) mice. On PND7, the lungs of *ERK1^+/+^* or *ERK1*^-/-^ mice exposed to either 21% O_2_ (normoxia) or 70% O_2_ (hyperoxia) from PND1 to PND7 were collected for the analyses of inflammatory genes. (**A**–**F**) RT-PCR analyses-based determination of *CCL2* (**A**), *CCL3* (**B**), *ICAM-1* (**C**), *IL-1β* (**D**), *IL-10* (**E**), and *TNF-α* (**F**) mRNA levels. Values are presented as mean ± SD (*n* = 4–7/genotype/exposure). Significant differences between genotypes and exposures are indicated by *, *p* < 0.05, **, *p* < 0.01, ***, *p* < 0.001, and ****, *p* < 0.0001 (ANOVA). ns = not significant.

Global ERK1 deficiency does not potentiate hyperoxia-induced lung inflammation in neonatal mice: Lung inflammation was evaluated by quantifying the mRNA levels of the pro-inflammatory cytokines (chemokine (C-C motif) ligand [CCL] 2, CCL3, intercellular adhesion molecule [ICAM]-1, interleukin [IL]-1β, and tumor necrosis factor [TNF]-α) and anti-inflammatory cytokine (IL-10) in lung tissues. Hyperoxia increased the expression of pro-inflammatory cytokines, CCL2 (Figure 8A), CCL3 (Figure 8B), and ICAM-1 (Figure 8C), and decreased the mRNA level of the anti-inflammatory cytokine, IL-10 (Figure 8E) without affecting the expression of the pro-inflammatory cytokines, IL-1β (Figure 8D) and TNF-α (Figure 8F) in our model. However, the effects of hyperoxia on the mRNA levels of these cytokines were comparable between ERK1^-/-^ and ERK1^+/+^ mice (Figure 8A–F), indicating that isolated ERK1 deficiency does not potentiate or attenuate hyperoxia-induced lung inflammation in neonatal mice.

**Figure 9:**
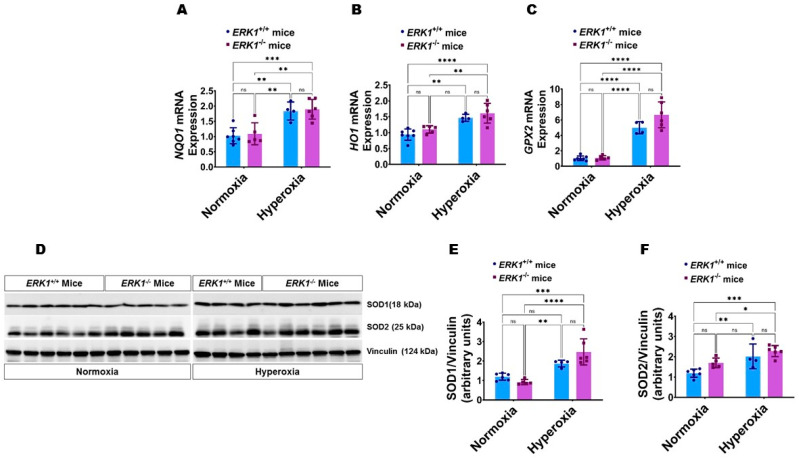
Lung antioxidant enzyme expression in global *ERK1*-sufficient (*ERK1^+/+^*) and -knockout (*ERK1*^-/-^) mice. On PND7, the lungs of *ERK1^+/+^* or *ERK1*^-/-^ mice exposed to either 21% O_2_ (normoxia) or 70% O_2_ (hyperoxia) from PND1 to PND7 were collected for quantifying the expression of antioxidant enzymes. (**A**–**C**) RT-PCR analyses-based determination of *NQO1* (**A**), *HO1* (**B**), and *GPX2* (**C**) mRNA levels. (**D**) Immunoblot determination of SOD1, SOD2, and vinculin protein levels. E-F Densitometric analysis wherein SOD1 (**E**) and SOD2 (**F**) band intensities were quantified and normalized to vinculin. Values are presented as mean ± SD (*n* = 4–7/genotype/exposure). Significant differences between genotypes and exposures are indicated by *, *p* < 0.05, **, *p* < 0.01, ***, *p* < 0.001, and ****, *p* < 0.0001 (ANOVA). ns = not significant.

Global ERK1 deficiency does not potentiate hyperoxia-induced oxidative stress in neonatal mice: We estimated oxidative stress in our model by analyzing the expression of the well-known antioxidant enzymes, NAD(P)H quinone dehydrogenase 1 (NQO1), heme oxygenase 1 (HO1), glutathione peroxidase 2 (GPX2), superoxide dismutase (SOD) 1, and SOD2 in lung tissues by real-time RT-PCR analysis and immunoblotting. Hyperoxia increased the mRNA levels of NQO1 (Figure 9A), HO1 (Figure 9B), and GPX2 (Figure 9C), and protein levels of SOD2 (Figure 9D,F); however, the effects of hyperoxia on the mRNA and protein levels of these enzymes were comparable between *ERK1*^-/-^ and *ERK1^+/+^* mice (Figure 9A–D,F). Additionally, hyperoxia significantly increased SOD1 protein levels in ERK1-deficient but not ERK1-sufficient mice; however, there was no significant difference in SOD1 protein levels between the genotypes in normoxic or hyperoxic conditions (Figure 9D,E). These findings suggest that isolated *ERK1* deficiency does not potentiate or attenuate hyperoxia-induced oxidative stress in neonatal mice.

## 4. Discussion

In this study, we investigated the effects of *ERK1* deficiency on human neonatal lung microvascular endothelial cell tubule formation and redox homeostasis in vitro and those of global *ERK1* deficiency on hyperoxic lung injury in neonatal mice in vivo. Our in vitro experiments suggest that *ERK1* deficiency alone is not necessary for both lung angiogenesis and maintaining pulmonary redox homeostasis. Further, our in vivo experiments indicate that global *ERK1* deficiency alone does not augment alveolar and pulmonary vascular simplification, inflammation, and oxidative stress in hyperoxia-exposed neonatal murine lungs.

Lung endothelial cell health plays a vital role in maintaining lung health across the life span in both humans and rodents. In BPD infants, lung angiogenesis is disrupted [8,9,10,12,39]. Similarly, in rodents with experimental BPD, the structurally and functionally abnormal lung vasculature promotes alveolar simplification and lung dysfunction and delays the resolution of neonatal lung injury [1,11,13,14,15,16,40]. These findings emphasize that lung endothelial cells are important in BPD pathogenesis and, thereby, are important therapeutic targets to improve the mortality and morbidities of BPD infants. However, the mechanisms through which these cells contribute to BPD pathogenesis remain unclear and are the focus of many preclinical and translational studies. Our previous studies demonstrate that pharmacological inhibition of *ERK1/2* signaling [27] and genetic inhibition of *ERK2* signaling [28] decrease HPMEC angiogenesis in vitro. We also demonstrated that deficient *ERK2* signaling in the endothelial cells potentiates experimental BPD and PH [28]. However, whether *ERK1* and *ERK2* have distinct or redundant functions in developing lungs remains unclear. Therefore, we sought to investigate the mechanistic role of *ERK1* signaling in BPD. Initially, we investigated the relative expression of ERK1 and ERK2 protein levels in the human lung microvascular endothelial cells. Consistent with the existent literature [41,42], we observed that levels of ERK2 protein are greater than those of ERK1 in these cells at baseline. We also observed a similar finding when these cells were exposed to hyperoxia. Further, *ERK1* inhibition did not exert an antiangiogenic effect *in vitro*. Additionally, we observed a similar finding *in vivo*. Global *ERK1* deficiency in neonatal mice did not decrease lung angiogenesis at baseline and following hyperoxia exposure. Several studies [43,44], including ours [27], have demonstrated that inhibition of both *ERK1* and *ERK2* decreases angiogenesis. We have also shown a similar antiangiogenic effect when *ERK2* signaling is inhibited [28]. However, this study is one of the few studies to investigate the effect of ERK1 signaling on lung endothelial cell heath. Our in vitro and in vivo findings suggest that isolated *ERK1* deficiency does not have a vascular phenotype, which is consistent with the findings by Pages and colleagues [45]. In a recent study by Ricard et al. [46], isolated endothelial *ERK1* deficiency did not affect arteriogenesis, whereas global *ERK1* deficiency or combined endothelial and macrophage *ERK1* deficiency resulted in increased but dysfunctional arteriogenesis in a mouse model of acute hindlimb ischemia. They observed increased macrophage infiltration at the arteriogenesis site after inducing ischemia. Their findings indicate that *ERK1* may interact with other cells and affect vascular health in pathologic states. However, we did not observe increased lung inflammation or lung angiogenesis after neonatal exposure in global *ERK1* deficient mice. Some of the reasons for these discrepant findings may be due to the differences in the nature of the insult, organs studied, and the developmental stage of mice. Our recent and current findings suggest that *ERK2* may play a major role than *ERK1* in maintaining lung endothelial cell health, both at baseline and under pathological conditions.

Hyperoxia causes experimental and human BPD by affecting pathways necessary for lung development and repair [47,48]. Further, the lung phenotype of our hyperoxia mouse model resembles human BPD [31,32]. So, we investigated if *ERK1* signaling regulates hyperoxic lung injury using global *ERK1* deficient mice. Kim et al. [49] showed that the formyl peptide receptor 2 agonist, WKYMVm hexapeptide-mediated ERK1/2 activation mitigates hyperoxia-induced neonatal lung injury in mice. We also recently showed that endothelial *ERK2* deficiency potentiates hyperoxic lung injury in neonatal mice [28]. However, Zhang et al. [50] and Carnesecchi et al. [51] noted an opposite effect of ERK1/2 activation on hyperoxia-induced lung injury in adult rodents. A similar finding of potentiation of hyperoxic lung injury due to ERK1/2 activation was noted in neonatal rodents, mainly mediated through fibroblasts [52,53]. Differentiation of alveolar interstitial fibroblast into lipofibroblast is necessary for lung development [54,55], whereas their differentiation into myofibroblast inhibits alveolarization [55]. These discrepant findings may be due to differences in the cell type and organ studied, activation of *ERK1* and *ERK2* isoforms, and the interactions between ERK1/2 and other pathways. Therefore, we investigated the effects of the *ERK1* isoform and found that isolated deficiency of this isoform does not potentiate hyperoxia-induced alveolar simplification in neonatal mice. Additionally, *ERK1* deficiency did not potentiate hyperoxia-induced lung inflammation in neonatal mice. Finally, we determined the effect of isolated *ERK1* deficiency on hyperoxia-induced oxidative stress because the latter is one of the major BPD risk factors [56,57]. We determined oxidative stress in vivo indirectly by quantifying the expression of antioxidant enzymes since it is difficult to quantify the highly unstable reactive oxygen species in real time. Our findings indicate that, unlike endothelial *ERK2* deficiency, isolated deficiency of *ERK1* does not influence the hyperoxia-induced expression of antioxidant enzyme expression in neonatal mice. We also show that *ERK1* knockdown does not affect hyperoxia-induced H_2_O_2_ generation in HPMECs. Collectively, the findings from our recent [28] and current studies highlight that *ERK2* rather than *ERK1* plays a mechanistic role in the pathogenesis of hyperoxia-induced neonatal lung injury. It is possible that increased *ERK2* activation in global *ERK1* knockout mice may have protected them from worsening lung injury when exposed to hyperoxia because the outcomes may be ultimately dependent on the total ERK1/2 activity, as demonstrated recently. In an elegant study, Fremin et al. [41] demonstrated that the severe developmental defects seen in *ERK2*-knockout mice can be rescued by increasing the total ERK1/2 activity from the transgenic expression of *ERK1*. These findings indicate that the phenotype positively correlates with the total ERK1/2 activity, highlighting the functional redundancy of ERK1 and ERK2. In our future studies, we will use genetic approaches to increase *ERK1* expression in *ERK2*-deficient endothelial cells to further differentiate the redundant and distinct functions of *ERK1* and *ERK2* in neonatal hyperoxic lung injury. It is also possible that *ERK1* may regulate neonatal lung injury in a sex-specific manner. Additionally, *ERK1* may preferentially affect lung function rather than lung development. Finally, *ERK1* may have a pathogenic role in developmental lung injury mediated by other insults, including lipopolysaccharide. We plan to address these gaps in our future studies. Nevertheless, we used robust genetic approaches using both human pulmonary microvascular endothelial cells and neonatal mice and performed rigorous studies to determine the mechanistic role of *ERK1* signaling in hyperoxia-induced neonatal lung injury.

In summary, we demonstrate that isolated *ERK1* signaling is not required for HPMEC tubule formation and, thereby, probably for human lung angiogenesis. We also show that *ERK1* is not a major regulator of hyperoxia-induced oxidative stress in these cells. Further, our in vivo studies demonstrate that global *ERK1* deficiency is dispensable for hyperoxia-induced neonatal murine lung injury. To the best of our knowledge, this is the first study to investigate the role of isolated *ERK1* signaling in hyperoxia-induced experimental BPD. Our findings reinforce that targeting *ERK2* rather than *ERK1* may improve BPD outcomes.

## 5. Conclusions

*ERK1* is dispensable for hyperoxia-induced experimental BPD due to compensatory ERK2 overactivation.

## Data Availability

The data presented in this study are available in the article.
